# Tunable WGM Laser Based on the Polymer Thermo-Optic Effect

**DOI:** 10.3390/polym13020205

**Published:** 2021-01-08

**Authors:** Shuai Zhang, Tianrui Zhai, Libin Cui, Xiaoyu Shi, Kun Ge, Ningning Liang, Anwer Hayat

**Affiliations:** College of Physics and Optoelectronics, Faculty of Science, Beijing University of Technology, Beijing 100124, China; zhangshuai@emails.bjut.edu.cn (S.Z.); clb0910@163.com (L.C.); xyshi@bjut.edu.cn (X.S.); GEKUN@emails.bjut.edu.cn (K.G.); liangnn@iccas.ac.cn (N.L.); anwerhayatnoor@gmail.com (A.H.)

**Keywords:** continuously tunable, whispering-gallery-mode laser, thermo-optic effect, the two-ring coupling effect

## Abstract

In this work, the thermo-optic effect in polymers was used to realize a temperature-tunable whispering-gallery-mode laser. The laser was fabricated using a capillary tube filled with a light-emitting conjugated polymer solution via the capillary effect. In the whispering-gallery-mode laser emission wavelength can be continuously tuned to about 19.5 nm using thermo-optic effect of polymer. The influence of different organic solvents on the tuning rate was studied. For a typical lasing mode with a bandwidth of 0.08 nm, a temperature-resolved tuning rate of ~1.55 nm/°C was obtained. The two-ring coupling effect is responsible for the suppression of the WGM in the micro-cavity laser. The proposed laser exhibited good reversibility and repeatability as well as a sensitive response to temperature, which could be applied to the design of photothermic and sensing devices.

## 1. Introduction

In the recent years, optical microcavities with various geometries have received significant attention, including Fabry–Perot structures [1,2], photonic crystals [3,4], distributed feedback structures [5,6,7], distributed Bragg reflectors [8,9], and whispering-gallery-mode (WGM) cavities [10,11,12,13]. The WGM cavity is small in size, has a very large quality (Q) factor, and exists in various geometries, including microspheres [14,15,16,17], microdisks [18,19,20,21], microrings [22,23], and microcylinders [24,25,26]. The microcylinder cavity can be realized in the capillary tube due to its easy fabrication process and low cost. In the field of microlasers, a unique type of capillary tube cavity possesses significant features, including perfect geometry, high flexibility, and ultra-high Q factors [27,28,29]. The cavity of silica capillary tubes can naturally handle liquid solutions, making it convenient for application as a microlaser cavity or sensor chamber. However, it is very difficult to realize tunability in such laser devices because of the good confinement of capillary tubes. Nonetheless, the conjugated polymer exhibits high absorbance, a high quantum efficiency [30,31], and a wide luminescence tuning range [32,33]. The strong thermo-optic effect of polymers is beneficial for fabricating tunable devices with a wide tuning range [34]. Therefore, polymer WGM lasers with a large tunable range can be achieved by combining the advantages of the high Q factors of capillary tubes and the strong thermo-optic effect of polymers. 

WGM laser devices are formed by capillary tubes, which is based on the total internal reflection of the light emitted by the gain medium along the curved interface between the high refractive index (RI) material, which is frequently a silica fiber [35], or capillary [36], and the low RI surrounding environment, normally formed by a conjugated polymer solution. The evanescent waves of the WGMs that resonate close to the curved interface of the microcylinder overlap with the gain solution around it and produce WGM laser emission. For this case, the RI of the microcylinder must be higher than that of the surrounding medium. Frequently, silica capillaries (RI = 1.45 in the visible range) are used as the ring resonator which possess ultrahigh Q factors. As a result, the WGM laser has high sensitivity to the subtle change of surrounding environment. The conjugated polymers have unique thermo-optic effect [37,38]. Zhao et al. developed a tunable WGM microlaser based on dye-doped cholesteric liquid crystal microdroplets with controllable size in an aqueous environment [39]. Dong et al. investigated thermal effects in Polydimethylsiloxane (PDMS) microspheres, and demonstrate their potential for highly sensitive thermal sensing [40]. These results not only shed light on the good tunability of lasing devices but also open up an avenue for the design of new sensing devices. 

Here, we present a continuously tunable WGM laser fabricated using a liquid-polymer-filled capillary tube. The device was prepared by filling a polymer solution into the capillary tube microcavity. The sensitive thermo-optic effect allowed the lasing wavelength range of the WGM laser to be tuned by up to 19.5 nm as the temperature increased from 24.5 to 39.1 °C. The maximum temperature-dependent tuning rate was up to 1.55 nm/°C between different organic solvents. Furthermore, we analyzed the two-ring coupling effect of the capillary-tube laser, which caused suppression of the WGM in the microcavity. The laser exhibited a good reversible response to changes in the ambient temperature. 

## 2. Fabrication of the Device 

**Figure 1:**
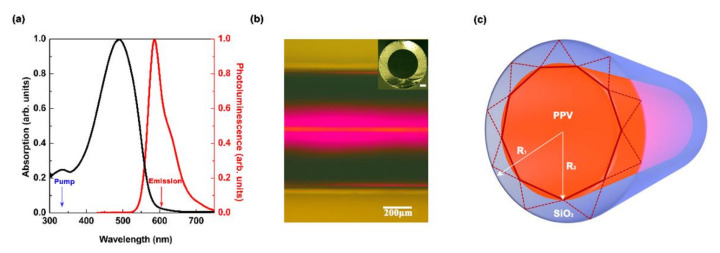
(**a**) Normalized photoluminescence (PL) spectrum (black) and absorption spectrum (red) of the conjugated polymer MDMO-PPV. The pump and emission wavelengths are shown by the blue and red arrows, respectively. (**b**) The optical micrograph of the device, with a scale bar of 200 μm. The inset is the cross section of the capillary tube and the scale bar is 100 μm. (**c**) Schematic diagram of the whispering-gallery-modes (WGMs) origin in capillary tube microcavity. There are two split rays caused by reflection/refraction, which are denoted by solid (dotted) lines.

First, a conjugated polymer poly[2-methoxy -5-(3′,7′-dimethyloctyloxy)-1,4-phenylenevinylene] (MDMO-PPV, Sigma-Aldrich) MDMO-PPV was dissolved in xylene at a concentration of 8.5 mg/mL. Figure 1a displays the absorption spectrum and the photoluminescence (PL) spectrum of the conjugated polymer MDMO-PPV. Nonpolarized white light from a tungsten halogen lamp (HL-2000) was used to characterize the absorption spectrum. There was a little overlap between the absorption and the PL spectrum, indicating that self-absorption of the PL emission was very weak. The wavelengths of the pump and emission are denoted by the blue and the red arrows, respectively, as shown in Figure 1a. Then, the organic polymer solution was imbibed into a silica capillary tube via the capillary effect. Finally, the sample was dipped in a solution composed of polyvinyl alcohol (PVA, Sigma-Aldrich, St. Louis, MO, USA) dissolved in deionized water at a concentration of 40 mg/mL, which acted as a sealant. Figure 1b shows an optical micrograph of the capillary-tube liquid-polymer WGM laser. The inset is the cross section optical micrograph of the capillary tube and the scale bar is 100 μm. The origin of mode in the WGM laser of the capillary-tube liquid-polymer is shown in the schematic diagram of Figure 1c. R_1_ is the outer radius of 300 μm and R_2_ is the inner radius of 225 μm. The modulation between the two sets of the WGM was caused by the interfaces of two media, as shown in Figure 1c, which will be discussed in detail later.

## 3. Experiment Results and Discussion

### 3.1. WGM Lasing Properties

**Figure 2:**
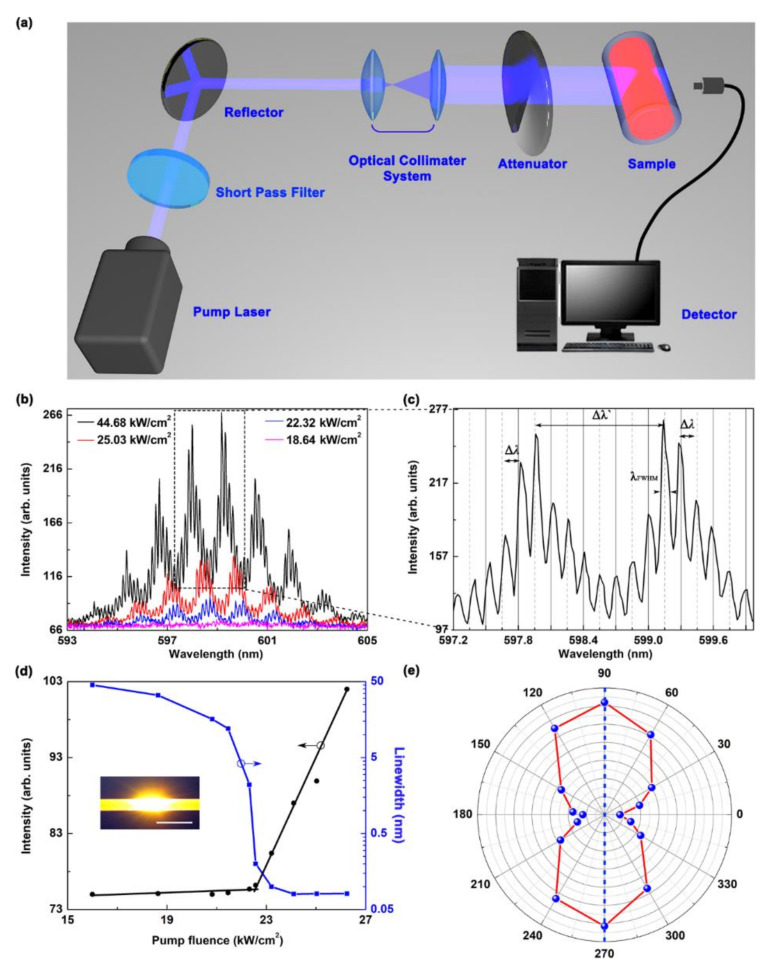
Lasing spectrum and emission properties of the capillary-tube liquid-polymer WGM laser at room temperature (24.5 °C). (**a**) Schematic optical layout of the measuring capillary-tube liquid-polymer WGM laser. (**b**) The lasing spectrum of the WGM laser collected at the increased pump Intensity. (**c**) Enlarged view of the WGM lasing mode clusters. The insets are the simulation of the WGM distribution. (**d**) The output peak intensities (the black circle with linear fitting) and linewidths (blue squares connected with a line) of one lasing peak as a function of the pump energy. Insets: Photograph of the excited WGM laser, with a scale bar of 2 mm. (**e**) Polarization-dependent lasing intensities of the WGM laser.

The WGM lasing spectrum and emission properties are presented in Figure 2. Figure 2a presents the optical layout for measuring the emission spectra. The short-pulsed diode-pumped solid-state laser (343 nm, 1 ns, 400-Hz repetition rate, Coherent Inc., Santa Clara, CA, USA) is employed as a pump source. The pumping laser spot was modified by the optical collimated system to ensure that the pump beam covered the entire device. The short-pass filter was used to remove second harmonic generation in the pump laser. The power of the pump source was regulated by a variable neutral optical attenuator. Spectroscopic characterization of the WGM laser was carried out using an optical spectrometer (HR 4000, Ocean Optics) with a resolution of ~0.03 nm.

Figure 2b shows the high-resolution spectra collected at different pump intensities, and sharp peaks are observed in the output spectra of the WGM laser. As the pump energy was increased above 22.3 kW/cm^2^, lasing peaks were observed in the emission spectrum. The output intensity increased significantly with increasing pump intensity. The intensity of the peaks increased and gradually saturated with increasing pump intensity. An enlarged view of the peak spacing at a pump intensity of ~44.68 kW/cm^2^ is shown in Figure 2c.

The spacing between two adjacent peaks Δ*λ* was almost the same at ~0.15 nm. The free spectral range Δ*λ* can be expressed as [24]:

(1) $$\Delta \lambda =\frac{\lambda^{2}}{L_{1}}$$

where λ is the emission wavelength and *L*_1_ is the effective optical path of the inner microcavity. *L*_1_ *=* 2*n_PPV_* π*R*_1_, where the refractive index of the MDMO-PPV xylene solution *n_PPV_* is ~1.71, which was similar to the experimental value of the MDMO-PPV xylene solution (1.73).

Note that there were several dips in the emission spectra, forming some mode clusters. The lasing modes in the dips were suppressed because of the two-ring coupling effect. In other words, a suppressive action occurred between the two nearby ring cavities. As shown in the Figure 1c, there are two sets of the WGMs supported by two ring cavities in the capillary tube microcavity, respectively. It can be regarded as a dimer of two ring cavities with gain and loss. The WGMs supported by both ring cavities experience an additional loss due to the mode leakage from the gain cavity (inner ring) to the lossy cavity (outer ring). It is the origin of the dips in Figure 2b.

The spacing between adjacent mode clusters Δ*λ*′ can be calculated as:

(2) $$\Delta \lambda^{\prime }=\frac{\lambda^{2}}{L_{2}-L_{1}}$$

where *L*_2_ is the effective optical path of the outer silica microcavity. However, *L*_2_ is determined by the refracted ray path. Under the condition 2*n_SiO_2__*π*R*_1_ < *L*_2_< 2*n_SiO_2__*π*R*_2_, the calculated spacing between adjacent mode clusters is Δλ′ = 1.17 nm.

The emission intensity and the linewidth as a function of the pumping energy are presented in Figure 2d. The related image showing lasing from the liquid polymer WGM laser is presented in the inset of Figure 2d. We also found that the polarization characteristics of WGM lasing was of the transverse electric mode (TE), which is shown in Figure 2e. TE mode lasing was obtained because of the high reflective coefficient. The lasing polarization vector was along the axial direction of the device.

**Figure 3:**
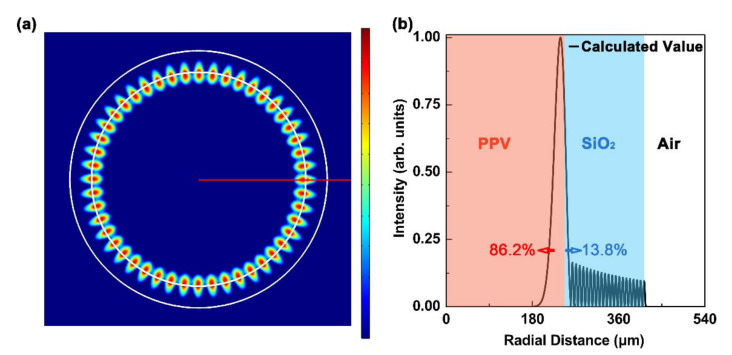
Simulated electric field distribution (transverse-electric mode (TE_0_)) of the capillary-tube liquid-polymer cavity. (**a**) Top view of the electric field distribution of the laser cavity. The white circles denoted the boundaries of the capillary tube. (**b**) Simulated intensity profile (TE_0_) of the capillary tube cross section along the red line in (**a**).

The electric field distribution of the laser device is studied using the COMSOL software based on the finite element method, as shown in Figure 3. Only the fundamental transverse-electric mode (TE_0_) can be excited in our proposed cavity. The electric field is well confined in the cavity which formed by silica and liquid polymer, as can be clearly seen in Figure 3a. Figure 3b exhibits the simulated intensity profile of the capillary tube cross section along the red line in Figure 3a. For the TE_0_ mode, the mode confinement in the PPV layer exceeds 86%. Correspondingly, the amount of light spreads into the SiO_2_ layer is less than 14%.

### 3.2. Continuously Tunable Characteristics

**Figure 4:**
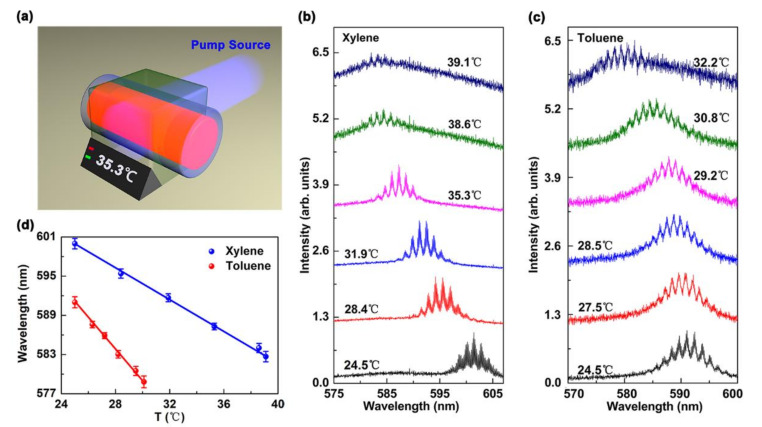
Performance of the continuously tunable capillary-tube liquid-polymer WGM laser. (**a**) Schematic of the heating platform equipment. (**b**,**c**) Varying emission spectrum for MDMO-PPV dissolved in xylene or toluene as a function of temperature (*T*). (**d**) Device emission wavelength tuning range for the devices with different organic solvents, i.e., xylene (blue spheres and the fitting line) and toluene (red spheres and the fitting line).

The lasing spectrum of the proposed WGM laser was measured as a function of temperature. The schematic of the heating system is shown in Figure 4a. Figure 4b presents the lasing spectrum for MDMO-PPV dissolved in xylene. The emission wavelength varied from 601.4 to 581.9 nm, as the temperature increased from 24.5 to 39.1 °C. Additionally, we investigated two organic solvents to study their influence on the tuning range of the emission wavelength. In Figure 4c, for MDMO-PPV dissolved in toluene, the lasing spectrum varied from 591.1 nm to 579.1 nm as the temperature increased from 24.5 to 32.2 °C.

Figure 4d shows a comparison of the liquid-polymer WGM laser in two solvents with a wide tuning range of ~19.5 nm and a maximum tuning rate is 1.55 nm/°C. The limit of tunablility (LOT) can be expressed as:

(3) $$LOT=3.3\times \frac{Standard\;Deviation}{Tuning\;Rate}$$

The LOT indicates the minimum temperature variation can make a wavelength shift of the tunable laser. The standard deviation is 0.55 nm. The LOT of the capillary-tube liquid-polymer WGM laser is 1.17 °C.

Because of the thermo-optic effect of the polymer, the emission wavelength gradually shifted far from the center of the PL spectrum with increasing ambient temperature. In this case, the luminous efficiency of the polymer reduced along with a gradual decrease in the lasing intensity. The thermally-induced resonant wavelength shift *δλ* can be expressed as follows [41,42,43]:

(4) $$\delta \lambda =\lambda_{0}\left(\frac{1}{n}\frac{dn}{dT}\Delta T+\frac{1}{D}\frac{dD}{dT}\Delta T\right)$$

where $\frac{dn}{dT}$ denotes the thermo-optic coefficient and $\frac{1}{D}\frac{dD}{dT}$ indicates the thermal expansion coefficient. *λ*_0_ designates the cavity resonant wavelength at room temperature and Δ*T* denotes the temperature change of the capillary-tube liquid-polymer microcavity. The thermo-optic coefficient ($\frac{dn}{dT}$) for devices with the polymer dissolved in xylene and toluene solutions are −3.0 × 10^−3^ and −4.3 × 10^−3^ K^−1^, respectively. The geometric parameters of the silica capillary tube were almost unchanged with varying temperature. In this work, the contribution of $\frac{1}{D}\frac{dD}{dT}$ was negligible (10^−6^ K^−1^).

As is shown in Table 1, the mean value and standard deviation (σ) of emission wavelength shift are 18.24 and 0.56 nm, respectively, which showed the stability of the continuously tunable property of the proposed lasing devices.

**Figure 5:**
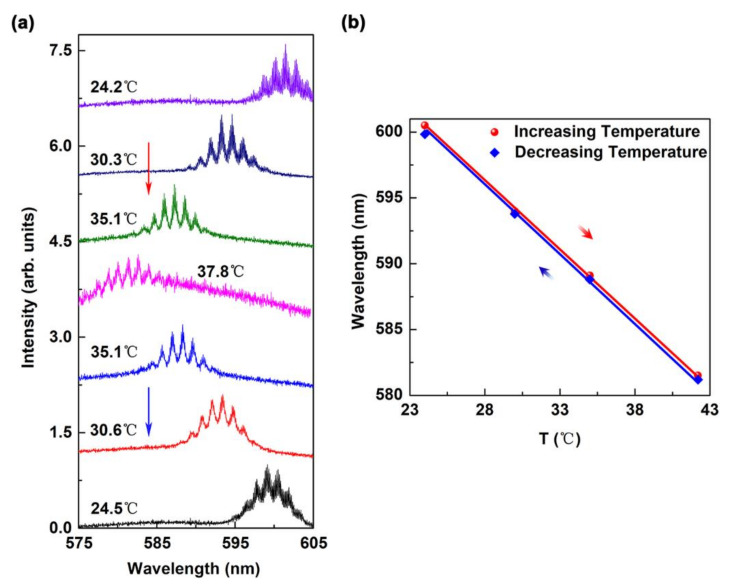
Reversibility of the continuously tunable WGM laser device. (**a**) Lasing spectrum as a function of cycled ambient temperature. (**b**) The emission wavelength as a function of temperature. The blue symbols and line denote a decreasing temperature and the red symbols and line denote an increasing temperature.

We also investigated the reverse process for the continuously tunable WGM laser. Figure 5a shows the lasing spectra for the complete cycle of the ambient temperature. The lasing spectrum exhibited a continuous blue shift with increasing temperature, while there was a red shift with decreasing temperature. This reversible action was expected because the refractive index of the device varies with temperature. Figure 5b shows the lasing wavelength as a function of the ambient temperature for the cases with increasing and decreasing temperature. The results demonstrate that the lasing wavelength has a linear shift with temperature, which implies that the proposed capillary-tube liquid-polymer WGM laser has good wavelength reversibility.

### 3.3. Discussion

Our study provides a possible solution to realize a continuously tunable WGM laser device with the merits of low-cost and easy fabrication. The emission characteristics show excellent lasing performances. Further experiment indicates that the laser device possesses a good spectral reversibility while changing the temperature. The proposed capillary-tube based liquid-polymer WGM laser is capable of low threshold lasing which can be readily tuned by temperature, making it suitable for sensing in liquid samples or integrating into flexible photothermic devices, chemical analyzers. In the next step, we will explore to broaden the tuning range of the emission wavelength by optimizing polymer materials. The tuning rate of the laser device can also be investigated further.

## 4. Conclusions

A continuously tunable WGM laser was demonstrated using a liquid-polymer microcavity in a capillary tube. Whispering gallery mode suppression was observed via two-ring coupling of the capillary-tube microcavity. The fundamental transverse-electric mode (TE_0_) can be excited in our proposed cavity and the mode confinement in the PPV layer exceeds 86%. Based on the thermo-optic effect of the liquid-polymer WGM laser, the lasing wavelength could be continuously tuned from 601.4 to 581.9 nm by adjusting the ambient temperature within a 14.6 °C interval. Different organic solvents were employed to explore the turning rate with varying ambient temperature. Furthermore, the experimental results indicated that the device had good reversibility with changing temperature, which makes the capillary-tube liquid-polymer WGM laser to be a promising candidate for highly sensitive temperature sensing in photothermic devices.

## Figures and Tables

**Table 1:** Emission wavelength of different devices with the function of increasing temperature.

	24 °C	30 °C	35 °C	42.2 °C	*δλ* (nm)
1	600.5	593.5	589	582.5	18
2	600.2	593	588.4	581.7	18.5
3	601.2	593.5	589.9	582.2	19
4	600	593	589.3	581.8	18.2
5	599	592.5	589	581.5	17.5
$\bar{\lambda }$ (nm)	600.18	593.1	589.12	581.94	18.24
σ	0.80	0.41	0.54	0.40	0.56
